# The Information Needs and Experiences of Cardiac Implantable Electronic Device Caregivers: A Qualitative Analysis of Social Media Data

**DOI:** 10.1007/s10880-026-10147-y

**Published:** 2026-04-06

**Authors:** Mitchell Nicmanis, Melissa Oxlad, Anna Chur-Hansen

**Affiliations:** School of Psychology, College of Education, Behavioural and Social Sciences, Adelaide University, Adelaide, Australia

**Keywords:** Cardiac implantable electronic devices, Pacemaker, Implantable cardioverter-defibrillator, Caregivers, Social media

## Abstract

Although cardiac implantable electronic devices (CIEDs) significantly improve physiological and mortality outcomes, both patients and their caregivers can experience considerable psychosocial distress. This study aimed to characterise the information needs and experiences of CIED caregivers discussed on a social media platform. Data were systematically collected using a Python-based script that filtered for CIED caregivers’ posts to the social media platform Reddit based on predefined keywords. Reflexive content analysis was used to analyse and describe the data. From 2138 posts collected, 190 posts from 151 caregivers were identified after filtering and screening. Included caregivers most often cared for fathers or husbands, who typically had pacemakers and a median age of 67 years (IQR = 30). Four categories described the main topics of caregivers’ discussions: *Concerns about the health of patients* (79% of users), *Managing and coping with patients’ lives and deaths* (75%), *The difficulties of being a caregiver* (62%), and *Engagement with healthcare* (41%). This study highlights that CIED caregivers using social media reported substantial burdens and challenges, adopting social media as a source of support. Given the parallels with caregivers of other patient populations, the findings demonstrate the need for healthcare guidelines, policies, and practices to support CIED caregivers.

**Supplementary Information:**

The online version contains supplementary material available at https://doi.org/10.1007/s10880-026-10147-y.

## Introduction

Cardiac implantable electronic devices (CIEDs), including permanent pacemakers (PPMs), implantable cardioverter-defibrillators (ICDs), and cardiac resynchronisation therapy (CRT) devices, are effective therapeutic interventions for the management of cardiovascular diseases such as arrhythmias, heart failure, and the prevention of sudden cardiac arrest (Al-Khatib et al., 2018; Glikson et al., 2021; Kusumoto et al., 2019; Shen et al., 2017; Zeppenfeld et al., 2022). CIEDs provide significant benefits for patients; however, evidence indicates that some recipients encounter psychosocial distress (Nicmanis et al., 2024b).

Although lifesaving, ICD implantation has been associated with elevated anxiety, depression, and post-traumatic stress symptoms (Ghezzi et al., 2023), and challenges in adapting to and living with the device (Barisone et al., 2022). These adverse psychosocial effects extend beyond patients. Their caregivers also face considerable burdens, with these roles associated with both familial and professional burnout (Gérain & Zech, 2021).

Research on CIED caregivers has primarily focused on the romantic partners of those with an ICD (Auld et al., 2021; Doolan-Noble et al., 2021; Dougherty et al., 2016; Rottmann et al., 2018; Streur et al., 2020; van den Broek et al., 2010; van den Broek et al., 2013). These caregivers experience interrelated physical and psychological changes with patients, and some come to experience distress, reporting changes in relationships, challenges in caregiving, and concerns about ICD shocks (Doolan-Noble et al., 2021; Dougherty et al., 2016; Rottmann et al., 2018; Schneider et al., 2020; Streur et al., 2020; van den Broek et al., 2010; van den Broek et al., 2013). Despite this, our understanding of the informational needs and experiences of caregivers, beyond romantic partners and across various CIED patient groups, remains limited.

Given the time and resource constraints that may limit caregivers’ participation in traditional research methods, the information they post to social media offers valuable insights into their experiences (Horrell et al., 2015). Caregivers frequently use social media to share experiences, seek support, and access caregiving information, positioning these platforms as a rich source of data for research (Bloom et al., 2019; Daynes-Kearney & Gallagher, 2023; Jacobs et al., 2016; Tuckey et al., 2022). These data offer access to caregivers’ candidly expressed perspectives as they occur in everyday life, capturing information they might not disclose to researchers or healthcare professionals (Smedley & Coulson, 2021).

This study aimed to characterise the information needs and experiences of CIED caregivers using data from a social media platform. The study employed a targeted data collection method to filter and identify caregivers’ posts in communities on the social media platform Reddit. Insights from this study can inform evidence-based recommendations for healthcare and aid in developing interventions for patients and CIED caregivers.

## Methods

### Design

This study used a qualitative design and adhered to the Standards for Reporting Qualitative Research (SRQR; Supplemental Table S1; O’Brien et al., 2014). Data were gathered from user-moderated thematically curated communities called “subreddits” hosted on the social media platform Reddit. The data consisted of posts from people who identified as the caregivers of patients with a CIED.

Reddit was chosen as it hosts communities where caregivers can discuss their experiences. Subreddits have been used extensively to investigate various healthcare topics (Proferes et al., 2021), including the experiences of people who are considering or have a CIED (Nicmanis et al., 2023, 2025b). Data were collected on November 18, 2024, from four subreddits (Supplemental Table S2) identified during a previous study (Nicmanis et al., 2023).

### Ethics

The University of Adelaide School of Psychology Human Research Ethics Sub-Committee approved this study (approval number: 22/27). Although all data collected were publicly available, protecting users and their online communities is imperative (Smedley & Coulson, 2021). Therefore, we excluded subreddits that prohibited data collection (if specified by user moderators in a subreddit’s rules), removed identifying information from reported extracts, and anonymised users using numeric pseudonyms.

### Data Collection

A Python script using the Python Reddit Application Programming Interface Wrapper was developed to collect social media posts made by the caregivers of patients with a CIED (Boe, 2023). First, the script systematically searched and collected posts from the identified subreddits using Reddit’s built-in search algorithm with predefined keywords related to CIEDs (Supplemental Table S3), informed by prior systematic reviews using device terms and an examination of terminology used by caregivers in online discussions. The script then deduplicated the collected posts and applied a keyword filter to remove posts that did not reference a predetermined list of caregiver roles (Supplemental Table S3). This approach minimised extraneous data from the identified subreddits that were unrelated to CIED caregiving activities. The script was validated using a test set of 15 posts from the four subreddits identified to verify that posts were accurately collected and appropriately deduplicated and filtered. The script automatically provided users with a numeric pseudonym.

Data collection was limited to initial caregiver posts, and did not include comment threads, to provide an overview of caregivers’ use of Reddit. While comments can provide additional context and interactional data, their inclusion would have substantially increased the volume of collected data and rendered manual analysis unmanageable. The number of posts collected was judged sufficient in line with the principle of information power given the novel focus of the study (Malterud et al., 2016), and additional posts did not add meaningful analytic value.

### Data Screening

The first author, a health psychology researcher and caregiver to a patient with a CIED, screened each collected post to exclude any remaining irrelevant posts. Posts were included if the text indicated that they were made by CIED caregivers, defined as unpaid individuals without formal training who assisted in managing various aspects of the lives of patients with a CIED whom they share a pre-existing emotional or cultural connection (e.g., family members, partners, friends, and community members; Castro et al., 2023).

During the screening process, a registry of user demographics was created based on the information provided in each post. The first author recorded each caregiver’s relationship to the patient. For every patient mentioned in the posts, age, gender, CIED type, duration of CIED implantation, and device indications were collected. Comprehensive demographic data for caregivers was often not provided in the posts.

### Analysis

Reflexive content analysis (Nicmanis, 2024) was used to systematically and inductively classify the data into codes, subcategories, and categories that represented topics of discussion on the subreddits related to the research question: “What are the information needs and experiences posted on social media by the caregivers of people with a CIED?” Data were initially coded based on their surface content and then iteratively refined by merging similar codes, subdividing vague ones, and correcting coding errors. The analysis drew on qualitative description as an interpretive process (Sandelowski, 2010) and critical realist research values (Maxwell, 2022) to create a reflexive qualitative description of the data. Frequencies of occurrence were calculated for the analysis based on the number of users who produced corresponding textual data.

The first author, conducted the analysis using the data analysis software NVivo 12 Plus. The second and third authors, experienced health psychologists with extensive expertise in cardiovascular care, guided and reviewed the analysis. Reported extracts from the data were revised for legibility. A detailed map of the qualitative research approach is presented in Supplemental Fig. S1.

## Results

### Data and Demographics

**Fig. 1:**
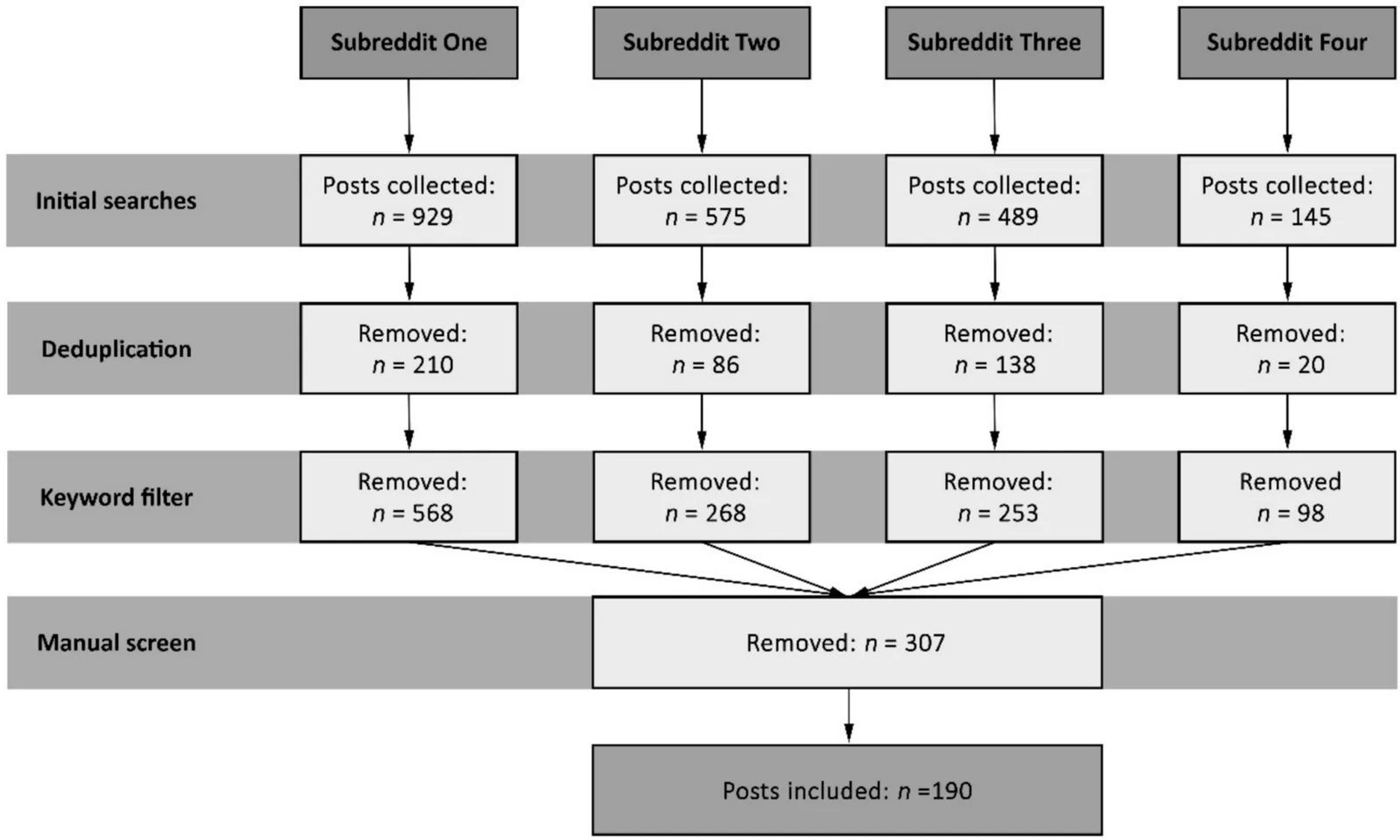
Data collection flowchart

The script collected 2138 posts. After deduplication, filtering, and manual screening, 190 posts were included in the analysis (Fig. 1). The included posts were made between 8 September 2015, and 17 November 2024. Most posts were made in the two years preceding data collection, with a median posting date of 29 September 2023.

The included posts were made by 151 CIED caregivers. Table 1 summarises caregivers’ relationships with patients and the demographic characteristics of those patients. The largest groups of caregivers cared for their fathers or husbands, who were typically implanted with a pacemaker and had a median age of 67 (*IQR* = 30).

**Table 1:** Demographics of patients and relation to caregivers

Characteristic	*n*	% of *N*
Patient status, *n* (%)		
Has a device	144	95%
Died with a device implanted	6	4%
Device removed	1	1%
Not reported	0	0%
Patient gender, *n* (%)		
Male	100	66%
Female	50	33%
Not reported	1	1%
Patient age (years), *n* (%)		
0–9	2	1%
10–19	2	1%
20–29	8	5%
30–39	9	6%
40–49	5	3%
50–59	10	7%
60–69	24	16%
70–79	24	16%
80–89	11	7%
90–99	3	2%
Not reported	53	35%
Relationship to patient, *n* (%)		
Father	52	34%
Mother	27	18%
Husband	22	15%
Wife	11	7%
Grandma	6	4%
Father-in-law	6	4%
Grandpa	6	4%
Partner	4	3%
Friend	4	3%
Brother	3	2%
Son	2	1%
Uncle	2	1%
Daughter	2	1%
Mother-in-law	2	1%
Sister	1	1%
Aunt	1	1%
Patient device type, *n* (%)		
PPM	76	50%
ICD	36	24%
ICD and PPM	23	15%
S-ICD	6	4%
CRT-D	6	4%
CRT	3	2%
Leadless PPM	1	1%
Patient time with device (years), *n* (%)		
< 1	57	38%
1–5	28	19%
5–10	11	7%
11–15	5	3%
16–20	3	2%
21–25	2	1%
26–30	1	1%
Not reported	44	29%
Device indication, *n* (%)		
Heart failure	30	20%
Prevention of sudden cardiac death	12	8%
Multi-cardiac comorbidities	12	8%
Heart block	10	7%
Bradyarrhythmias	8	5%
Tachyarrhythmias	7	5%
Atrial fibrillation	6	4%
Heart disease	6	4%
Genetic arrhythmia syndromes	3	2%
Ventricular fibrillation	1	1%
Chronic obstructive pulmonary disease	1	1%
Syncope	1	1%
Sick sinus syndrome	1	1%
Other specific conditions	4	3%
Not reported	49	32%

*Note*. Percentages based on number of respondents (*n*) and total sample size (*N* = 151). *CRT: * Cardiac Resynchronization Therapy; *CRT-D*: Cardiac Resynchronization Therapy Defibrillator; *ICD*: Implantable Cardioverter-Defibrillator; *PPM*: Permanent Pacemaker; *S-ICD* : Subcutaneous Implantable Cardioverter-Defibrillator

**Fig. 2:**
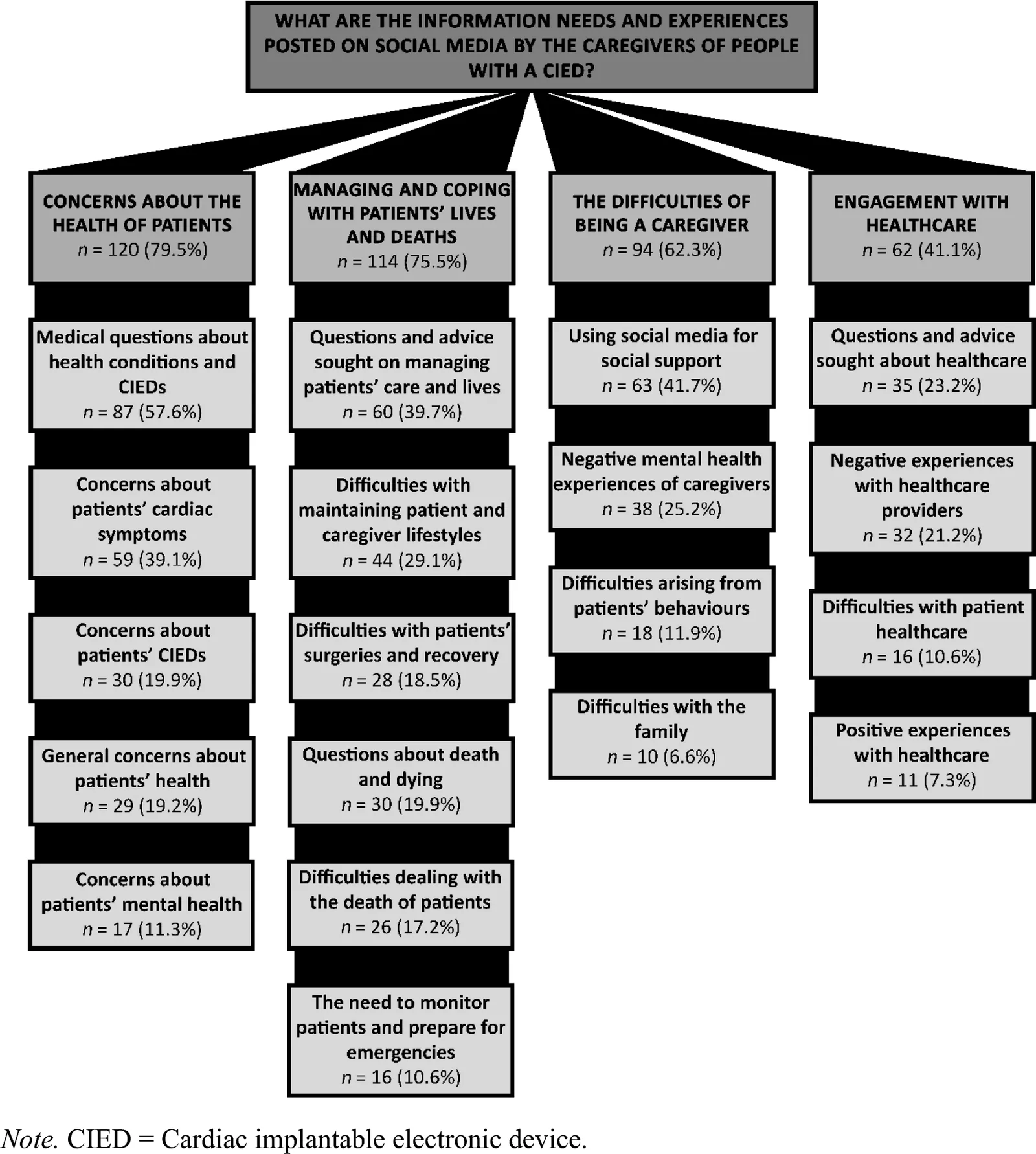
The analysis structure with categories in dark grey and subcategories in light grey with frequencies of occurrence calculated based on the number of users (*n*) that produced text supporting that category or subcategory and the total sample size (N = 151). *Note.* CIED = Cardiac implantable electronic device

The data were classified into 425 initial codes, which were refined into 174 codes. These codes were grouped into 19 subcategories and four overarching categories, to described the information needs and experiences of caregivers (Fig. 2; Supplemental Table S4).

Table 2 provides representative quotes from each subcategory. What follows is a narrative summary of the analysis, largely organized by category and subcategory frequency to reflect the relative salience of caregiver discussions on the platform.

**Table 2:** Representative quotes from caregivers for each subcategory

Categories and Subcategories	Example quote	*n* (% of *N*)
Concerns about the patient’s health		
Medical questions about health conditions and CIEDs	“He [caregivers’ Husband] is in the Heart hospital right now and they gave him this [URL link to image of medical test] …does the bottom part mean he flatlined?” (User 66)	87 (57.6%)
Concerns about patients’ cardiac symptoms	“They gave her [caregiver’s mother] a pacemaker 3 days ago and it hasn’t changed a thing, also the beta blockers are not reducing her heart rate. What more can they possibly do?” (User 10)	59 (39.1%)
Concerns about patients’ CIEDs	“It’s been 7 months post his [caregiver’s husband] ICD implantation… Are the leads intact and okay? What are the general signs if they are broken/damaged?” (User 344)	30 (19.9%)
General concerns about patients’ health	“He [caregiver’s husband] is constantly out of breath, his HR gets to 130 from doing simple tasks… We are so worried the ICD will not improve his quality of life as we had hoped” (User 358)	29 (19.2%)
Concerns about patients’ mental health	“Anything I can do to reassure her [caregiver’s mother] and make her less anxious? … She didn’t expect for a routine checkup to end up with the doctor saying they needed to replace one of the leads so that’s why she is kind of anxious.” (User 412)	17 (11.3%)
Managing the patient’s life with a CIED		
Questions and advice sought on managing patients	“I’m terrified about the stress on her [caregiver’s mother] heart … Should I convince her to go to the urgent care center?” (User 185)	60 (39.7%)
Difficulties with maintaining patient and caregiver lifestyles	“I know eventually we both have to go back to work, and do normal things but I’m terrified something will happen again.” (User 338)	44 (29.1%)
Difficulties with patients’ surgeries and recovery	“After the surgery, his [caregiver’s father-in-law] skin was sensitive and the doc said, it would take about a couple of months to heal completely. But now, his skin around the pacemaker are starting to turn grey and wrinkly.” (User 122)	28 (18.5%)
Questions about death and dying	“Is that all there is? Is there anything more to it than him [caregiver’s father] just being too weak? I know this explanation will change nothing, but I took care of him and ushered him through all of his healthcare for years, and this feels like an oversimplification.” (User 54)	30 (19.9%)
Difficulties dealing with the death of patients	“I’d never considered a situation where she [caregiver’s wife] might pre-decease me. I had thought she’d live to about 80, but how much is peripartum cardiomyopathy likely to reduce that by?” (User 101)	26 (17.2%)
The need to monitor patients and prepare for emergencies	“She [caregiver’s grandmother] wants to maintain her independence, but we worry about getting her help as fast as possible in the event that she goes into cardiac arrest with no one around. …Is there anything that monitors heart rate that when then send an alarm when the heart rate is consistent with cardiac arrest?” (User 33)	16 (10.6%)
The difficulties of being a caregiver		
Using social media for social support	“We are relieved and wanted to thank again all the community for all the knowledge, the encouragements, the experiences, you were our lifeline.” (User 350)	63 (41.7%)
Negative mental health experiences of caregivers	“My life is consumed with anxiety and stress of the what ifs. I know we can’t predict the future, but I can’t imagine if something would happen to him [caregiver’s husband].” (User 263)	38 (25.2%)
Difficulties dealing with the patient	“He’s [caregiver’s father-in-law] the kind of person who you kinda can’t tell anything, he doesn’t listen and refuses any help, but then moans no one helps/listens” (user 58)	18 (11.9%)
Difficulties with the family	“Since this happened, he [caregiver’s partner] fears that he’s just going to drop dead leaving me, our baby, his daughter, his stepson, and our 3 adopted children without a father.” (User 335)	10 (6.6%)
Engagement with healthcare		
Questions and advice sought about healthcare	“I’m honestly just wanting reassurance that what they are telling us is correct. If there is even a chance that she [caregiver’s mother] could have a continuous episode of vtach then I will take her to another hospital in the nearest city.” (User 9)	35 (23.2%)
Negative experiences with healthcare professionals	“I’m just praying that her [caregiver’s mother] healthcare team is taking her seriously, since as a person of color, her chances of poor outcomes are increased. I work in a hospital, and I see it from providers all the time, the way they treat certain patients.” (User 403)	32 (21.2%)
Difficulties with patient healthcare	“Her [caregiver’s grandma] next device appointment isn’t until June, and it’s hard to get a call back for non-urgent questions, so I thought I’d ask here for personal experiences while I wait to hear from them.” (User 267)	16 (10.6%)
Positive experiences with healthcare	“My cardiologist who is really an amazing doctor and a very rare find and also a very supportive doctor, he helped us a lot and is always available to take calls and clear our doubts …” (User 237)	11 (7.3%)

Frequencies of occurrence calculated based on the number of users (*n*) that produced text supporting that category or subcategory and the total sample size (N = 151). *CIED*: Cardiac implantable electronic device, *ICD*: Implantable Cardioverter-Defibrillator

### Concerns About the Health of Patients

#### Medical Questions About Health Conditions and CIEDs

Caregivers primarily discussed their concerns about patients’ health. Within this category, the most frequent subcategory captured caregivers’ medical questions about health conditions and CIEDs. Caregivers sought information on patients’ medical conditions, CIEDs, associated risks, and surgical procedures, including device and lead replacement, device deactivation, and supplementary surgeries. Additionally, some caregivers provided images of patients’ medical charts or descriptions of medical tests for interpretation by others.

#### Concerns About Patients’ Cardiac Symptoms

Closely related to these questions was the second subcategory, which described caregivers’ concerns about patients’ cardiac symptoms, including heart rate changes or arrhythmias, shortness of breath, diminished energy, dizziness or syncope, fluid retention, and blood pressure changes. Caregivers sought further information, and some expressed worries that CIED implantation had not improved patients’ cardiac outcomes.

#### Concerns About Patients’ CIEDs

The third subcategory described the specific concerns that caregivers had about patients’ CIED. These included concerns about ICD shocks and discomfort from device protrusion. Most commonly, caregivers were concerned about continued device function; including battery depletion, potential device damage, malfunctions, and device dependency.

#### General Concerns About Patients’ Health

The fourth subcategory reflected caregivers’ broader concerns about the health of patients. This included concerns about quality of life and physical comfort, as well as those related to medication side effects, non-CIED-related infections, and changes in weight.

#### Concerns About Patients’ Mental Health

The fifth subcategory captured concerns related to patients’ mental health. Caregivers discussed patients’ experiences of anxiety, fear, post-traumatic stress symptoms, cognitive declines, and stress. Caregivers described these experiences as challenging for both patients and themselves and sought advice on how to manage them.

### Managing and Coping with Patients’ Lives and Deaths

#### Questions and Advice Sought on Managing Patients’ Care and Lives

The second category captured caregivers’ discussions about managing patients’ lives and deaths with a CIED. The most frequent subcategory consisted of questions and advice sought on how to manage patients’ care and life with a device. Caregivers sought answers and guidance on supporting the patient with their device and associated issues, appropriate activities, and whether certain items were harmful to the patient. Other questions included ways to monitor the patient, support during emergencies, coping with psychological challenges, and various aspects of daily life with a CIED. Additionally, several caregivers used Reddit to determine whether emergency care was needed.

#### Difficulties with Maintaining Patient and Caregiver Lifestyles

The second subcategory captured difficulties discussed in maintaining patient and caregiver lifestyles. Caregivers expressed concerns about activities and lifestyle factors such as electromagnetic interference, travelling, sleep, diet, and physical activities that could harm the patient. Caregivers also discussed the lifestyle challenges of a device and the desire for them and their patients to maintain independence, employment, and a positive lifestyle.

#### Difficulties with Patients’ Surgeries and Recovery

The third subcategory detailed difficulties discussed by caregivers about patient surgery and recovery. They described concerns about patients undergoing surgeries and the potential risks involved. This included challenges in recovery, such as problems with the surgical implantation site, complications, and concerns about the device not being implanted correctly.

#### Questions About Death and Dying–Difficulties Dealing with the Death of Patients

The fourth and fifth subcategories, were closely interrelated, and captured caregivers’ questions about death and dying and their difficulties in dealing with patients’ death, Caregivers most often inquired about the likelihood of death, expected longevity, and how to prepare for death. Discussions also included questions about device deactivation and removal for end-of-life care and concerns about these processes. Following a patient’s death, caregivers reported struggling with their loss and requested further explanations.

#### The Need to Monitor Patients and Prepare for Emergencies

The sixth subcategory described caregivers’ need to monitor patients and prepare for emergencies. Caregivers discussed their concerns about emergency preparedness and the use of systems to monitor patients’ health. This included smartwatches and pulse monitors, as well as monitoring systems to help patients get assistance in case of emergencies, such as medical alert bracelets.

### The Difficulties of Being a Caregiver

#### Using Social Media for Social Support

The third category captured the difficulties of being a caregiver. The first subcategory reflected the use of the platform for social support to manage these difficulties. Caregivers sought reassurance and insight from others with similar experiences, and used the subreddit to obtain clear and relevant information not found elsewhere. Caregivers expressed thanks for this support, and some shared their own experiences to offer hope and information to others.

#### Negative Mental Health Experiences of Caregivers

The second subcategory summarized the negative mental health experiences of caregivers. Caregivers discussed feelings of anxiety, fear, stress, and emotional disturbance. Some expressed feelings of being lost or shocked, and these emotions were worsened by patients’ health declining or the sudden illness of previously healthy loved ones.

#### Difficulties Arising from Patients’ Behaviours

The third subcategory described the difficulties of being a caregiver that resulted from patients’ behaviours. Caregivers reported challenges in providing care or understand situations because they felt unable to trust patients to be truthful or accurately relay their symptoms. Difficulties also stemmed from patients’ resistance to seeing healthcare professionals or following medical advice. Some caregivers struggled with not wanting to pressure patients into treatment or compliance.

#### Difficulties with the Family

The last subcategory captured difficulties related to patients’ families. Caregivers discussed difficulties arising from other family members or caregivers, including temporary living arrangements and situations in which patients’ needs disrupted family functioning. Device implantation also shaped future planning for patients and caregivers, particularly among younger caregivers, who discussed issues such as family planning and concerns about a child’s future with a CIED.

### Engagement with Healthcare

#### Questions and Advice Sought About Healthcare

The final category described caregivers’ engagement with healthcare. The first subcategory captured the questions and advice caregivers sought about healthcare. Caregivers asked about healthcare professionals’ practices and decisions and sought second opinions or further details about patients’ conditions, often due to poor communication from providers. Questions were also asked about medical tests and organizing appointments.

#### Negative Experiences with Healthcare Providers

The second subcategory detailed caregivers’ perceived negative experiences with healthcare professionals. Caregivers were most often concerned that healthcare professionals had not acted in patients’ best interest or had caused harm. Others highlighted communication issues, citing examples of inadequate communication, conflicting opinions, and feeling that their own or the patient’s concerns were dismissed.

#### Difficulties with Patient Healthcare

The third subcategory captured caregivers’ discussion of border difficulties with patient healthcare. Accessing healthcare was challenging for caregivers due to logistical problems, financial constraints, and living in rural areas. Caregivers desired the best possible treatments and care and expressed concerns about the accuracy and safety of medical tests.

#### Positive Experiences with Healthcare

The last subcategory described caregivers’ positive experiences with healthcare. Some caregivers used the social media platform to express their gratitude towards healthcare providers, especially when they perceived their providers as providing a quality service or saving the patient’s life.

## Discussion

This study characterised the information needs and experiences of CIED caregivers shared on a social media platform. Caregivers primarily sought medical guidance, often reporting limited access to or understanding of essential information. In some cases, these gaps were substantial, such as when caregivers used the platform to assess the severity of patient symptoms and determine whether emergency medical care was necessary. Caregivers also described broader challenges, including managing the patient’s condition, adapting to lifestyle changes, and addressing concerns about healthcare professionals. Beyond the questions and advice sought on managing these challenges, caregivers used social media as a source of social support.

Previous research has largely focused on the quantitative evaluation of caregiver outcomes for patients with an ICD (Auld et al., 2021; Dougherty et al., 2016; Rottmann et al., 2018; Schneider et al., 2020; Streur et al., 2020; van den Broek et al., 2010; van den Broek et al., 2013). The findings of this study share parallels with the limited qualitative literature reporting the challenges experienced by ICD caregivers, including difficulties in obtaining health information, navigating relationship changes, coping with emotional stressors, and addressing issues related to device deactivation (Doolan-Noble et al., 2021; Fluur et al., 2014). There was limited discussion in the data of experiences with ICD shocks, despite previous research identifying these as a major source of concern and distress for caregivers (Fluur et al., 2014) and linking them to both patient (Ghezzi et al., 2023) and caregiver (Rottmann et al., 2018) outcomes. The limited discussion of shocks is likely due to the characteristics of the sample as the majority of caregivers cared for patients with a PPM. Given this, the findings provide insight into the experiences of patients with CIEDs other than ICDs and their caregivers, who have both received limited attention in the existing literature.

As the first study to examine CIED caregivers experiences using social media data, the findings show similarities to other online caregiver communities supporting patients with conditions such as cancer (Bloom et al., 2019), congenital abnormalities (Jacobs et al., 2016), chronic kidney disease (Tuckey et al., 2022), and caregivers more generally (Shoults et al., 2023). These parallels have important implications for healthcare policy and practice, as they highlight that the burdens common to caregivers of other serious conditions are also experienced by CIED caregivers. Therefore, as in other healthcare fields that have acknowledged caregivers as essential to patient care, this study highlights the need to strengthen support for these caregivers.

### Implications for Research and Practice

Existing guidelines developed by cardiovascular professional societies intended for healthcare professionals working with patients who have a CIED do not address caregiver needs beyond mentions in shared decision-making (Al-Khatib et al., 2018; Glikson et al., 2021; Kusumoto et al., 2019; Shen et al., 2017; Zeppenfeld et al., 2022). Only guidelines for end-of-life device care mention caregivers in detail (Lampert et al., 2010; Padeletti et al., 2010). The lack of acknowledgment of caregivers in CIED care is a critical oversight as the cost of informal caregiving for patients with cardiovascular diseases is projected to reach $128 billion in the United States alone by 2035 (Dunbar et al., 2018). While many countries have adopted national policies to acknowledge caregivers (e.g., the Caregiver Advise, Record, Enable Act in the United States), there are currently no healthcare guidelines or policies specifically addressing the psychosocial management of patients with a CIED or their caregivers.

This study demonstrated that caregivers experienced psychological distress and may possess limited knowledge of CIEDs and related patient care, as reflected in the nature of the questions posed by users. These findings align with previous research indicating a heightened risk of adverse outcomes among CIED caregivers (Doolan-Noble et al., 2021; Dougherty et al., 2016; Rottmann et al., 2018; Schneider et al., 2020; Streur et al., 2020; van den Broek et al., 2010; van den Broek et al., 2013). Given these issues, there are now growing calls for caregiver screening in cardiovascular care (Dunbar et al., 2018; Schneider et al., 2020).

As part of a patient-centred care approach, healthcare guidelines and policy should promote caregiver and patient screening to identify pressing concerns and coordinate interventions for those experiencing distress with the involvement of a multidisciplinary team (Dekker et al., 2023; Gaffey et al., 2022a, 2022b; Smolderen et al., 2024). Currently, no universal screening approach exists for patients or caregivers, and further research is needed, with recent guidelines for patients with cardiovascular disease recommending that screening protocols are developed in collaboration with qualified mental health professionals and tailored to work within local resources (Bueno et al., 2025). Further research should examine specialised interventions such as caregiver education programmes, peer-support, or psychological services. While education programs have had limited effects on patient-reported psychosocial outcomes among patients with a CIED (Nicmanis et al., 2025a), evidence suggests that involving partners in these interventions improves both patient and partner reported outcomes (Dougherty et al., 2019). However, further research is needed due to the methodological constraints of caregiver interventions in cardiovascular care, including inconsistent definitions of caregivers, heterogeneity in outcome measures, and limited examination of intervention timing (Knowles et al., 2021).

Guidelines and research must also consider improved communication with caregivers, as a concerning number in this study reported negative experiences with healthcare professionals, often due to perceived deficits in provider communication. As part of effective communication, shared decision-making should be at the forefront of guidelines, ensuring that caregivers are actively involved in conversations to comprehensively address the needs and concerns of both patients and their support networks (Chung et al., 2021). Communication should be approached from a multidisciplinary perspective, with professionals such as health psychologists, who have specialist expertise in biopsychosocial integrative care, playing an important role in bridging gaps between providers, caregivers, and patients (see Gaffey et al., 2022b).

In addition, this study reinforces the benefits of peer and social support for caregivers and patients with CIEDs (Nicmanis et al., 2023, 2025b; Rottmann et al., 2018). However, because Reddit content is community moderated, caregivers risk encountering cardiovascular-related misinformation (Farnood et al., 2022; Kostick et al., 2015). While formal, moderated web-based peer-support platforms exist, Reddit’s popularity suggests that current services may not meet caregivers’ needs (e.g., the desire to remain anonymous). Further research could draw on co-design principles to develop peer-support platforms that better address caregivers’ needs, while providing medically accurate information (O’Donnell et al., 2024).

Finally, this study underscores the need for improved end-of-life support for caregivers, as many had unanswered questions about death and dying. Family caregivers play a crucial role in device deactivation discussions (Hill et al., 2024). While guidelines for these discussions consider the role of caregivers (Lampert et al., 2010; Padeletti et al., 2010), evidence from a systematic review indicates that such discussions are often inconsistent or absent (Freemantle & Murtagh, 2022). Given the distress that bereavement can cause, research that develops interventions or tools for CIED caregivers may improve their outcomes (Nicmanis et al., 2024a). This could include the development of specific educational decision aids to facilitate informed decision-making for end-of-life care and device deactivation (Chen et al., 2025; Godfrey et al., 2025).

### Limitations

This study used a targeted data collection and filtering approach to identify posts from CIED caregivers on Reddit, offering insights into the naturalistically shared experiences and information needs of a group that is difficult to include in research. Although an extensive set of keywords was used to filter the posts, relying on specific terms may have inadvertently excluded relevant posts that did not contain those keywords. Additionally, data collected from Reddit is not representative of general clinical populations (Proferes et al., 2021). Given the nature of the platform, caregivers posting on Reddit are more likely to be technologically literate with access to the internet, and tend to be younger, male, and from English-speaking backgrounds (Amaya et al., 2019; Proferes et al., 2021). As the data were passively collected, further questions could not be asked of users, and therefore the accuracy of information in the data relied on caregivers’ own recounting of patient conditions, clinical indication, and experiences. Given this, the analysis was based on researcher interpretation of users’ posts and the inability to seek clarification from users may have affected the interpretation of intended meanings. This highlights the need for further in-depth qualitative studies with caregivers, such as semi-structured interviews or focus groups informed by the ideas identified in this study.

## Conclusion

The findings of this study indicate that CIED caregivers face challenges comparable to those seen in other caregiver communities on social media. This comparability highlights the need for healthcare guidelines, policies, and practices that provide robust support for CIED caregivers. This could include incorporating distress screening for CIED caregivers, implementing targeted caregiving interventions, enhancing communication with healthcare providers, establishing formal peer support networks, and improving support during end-of-life care. Together, these initiatives have the potential to substantially improve outcomes for both caregivers and their patients with a CIED.

## Supplementary Information

Below is the link to the electronic supplementary material.

Supplementary file1 (PDF 586 KB)

## Data Availability

Data are available on reasonable request to the corresponding author.
