# Supplementary material for: The Information Needs and Experiences of Cardiac Implantable Electronic Device Caregivers: A Qualitative Analysis of Social Media Data
Source: J Clin Psychol Med Settings. 2026 Apr 6;33(3):598–610. doi: 10.1007/s10880-026-10147-y (PMC13582173; doi:10.1007/s10880-026-10147-y)
Supplement: Supplementary file 1 [file 10880_2026_10147_MOESM1_ESM.pdf]

Supplemental Materials

Table S1

Standards for Reporting Qualitative Research (SRQR; O'Brien et al., 2014).

No. Topic	Item	Location where item is reported
**Title and abstract**		
S1 Title	Concise description of the nature and topic of the study identifying the study as qualitative or indicating the approach (e.g., ethnography, grounded theory) or data collection methods (e.g., interview, focus group) is recommended	See the title.
S2 Abstract	Summary of key elements of the study using the abstract format of the intended publication; typically includes objective, methods, results, and conclusions	See the Abstract.
**Introduction**		
S3 Problem formulation	Description and significance of the problem/phenomenon studied; review of relevant theory and empirical work; problem statement	See the final three paragraphs of the Introduction.
S4 Purpose or research question	Purpose of the study and specific objectives or questions	See the final paragraph of the Introduction and Data Analysis section of the method.
**Methods**		
S5 Qualitative approach and research paradigm	Qualitative approach (e.g., ethnography, grounded theory, case study, phenomenology, narrative research) and guiding theory if appropriate; identifying the research paradigm (e.g., positivist, constructivist/interpretivist) is also recommended	See the Data Analysis section and Supplementary Figure S1.
S6 Researcher characteristics and reflexivity	Researchers' characteristics that may influence the research, including personal attributes, qualifications/experience, relationship with participants, assumptions, or presuppositions; potential or actual interaction between researchers' characteristics and the research questions, approach, methods, results, or transferability	See the Data Analysis section and Supplementary Figure S1.
S7 Context	Setting/site and salient contextual factors; rationale	See Design and Data section.
S8 Sampling strategy	How and why research participants, documents, or events were selected; criteria for deciding when no further sampling was necessary (e.g., sampling saturation); rationale	See the Design and Data. Data Collection, and Data Inclusion and Exclusion sections. Also see Supplementary Figure S1.
S9 Ethical issues pertaining to human subjects	Documentation of approval by an appropriate ethics review board and participant consent, or explanation for lack thereof; other confidentiality and data security issues	See the Ethics section.

S10 Data collection methods	Types of data collected; details of data collection procedures including (as appropriate) start and stop dates of data collection and analysis, iterative process, triangulation of sources/methods, and modification of procedures in response to evolving study findings; rationale	See the Design and Data and Data Collection sections.
S11 Data collection instruments and technologies	Description of instruments (e.g., interview guides, questionnaires) and devices (e.g., audio recorders) used for data collection; if/how the instrument(s) changed over the course of the study	See the Data Collection section
S12 Units of study	Number and relevant characteristics of participants, documents, or events included in the study; level of participation (could be reported in results)	See the Data Collection and Demographics section.
S13 Data processing	Methods for processing data prior to and during analysis, including transcription, data entry, data management and security, verification of data integrity, data coding, and anonymization/deidentification of excerpts	See the Data Screening and Data Analysis sections.
S14 Data analysis	Process by which inferences, themes, etc., were identified and developed, including researchers involved in data analysis; usually references a specific paradigm or approach; rationale	See the Data Analysis Section and Supplementary Figure S1.
S15 Techniques to enhance trustworthiness	Techniques to enhance trustworthiness and credibility of data analysis (e.g., member checking, audit trail, triangulation); rationale	See the Data Analysis section and Supplementary Figure S1.
**Results/Findings**		
S16 Synthesis and interpretation	Main findings (e.g., interpretations, inferences, and themes); might include development of a theory or model, or integration with prior research or theory	See Analysis Structure section and Figure 2.
S17 Links to empirical data	Evidence (e.g., quotes, field notes, text excerpts, photographs) to substantiate analytic findings	See Table 2.
**Discussion**		
S18 Integration with prior work, implications, transferability, and contribution(s) to the field	Short summary of main findings; explanation of how findings and conclusions connect to, support, elaborate on, or challenge conclusions of earlier scholarship; discussion of scope of application/generalizability; identification of unique contribution(s) to scholarship in a discipline or field	See the Discussion section.
S19 Limitations	Trustworthiness and limitations of findings	See the Limitations section.
**Other**		
S20 Conflicts of interest	Potential sources of influence or perceived influence on study conduct and conclusions; how these were managed	See the Disclosures section.
S21 Funding	Sources of funding and other support; role of funders in data collection, interpretation, and reporting	See the Sources of Funding section.

Table S2.

Characteristics of Subreddits Included in Data Collection.

Subreddit Identifier	Description	Number of Members*
One	Intended for cardiac implantable electronic device patients and carers to discuss these devices.	2000+
Two	Intended for users to ask medical questions and have these answered by other users identified as medical professionals.	500,000+
Three	Intended for all users to ask questions about cardiology	4000+
Four	Intended for the discussion of living with heart failure by patients and caregivers.	4000+

Note. * = approximate number of members at time of data collection.

Table S3.

Keywords Used for Searches and Keyword Filtering.

Search Keywords	Filter Keywords
["cardiac contractility modulation", "cardiac implantable electronic device", "cardiac resynchronisation", "cardiac resynchronization", "CRT", "ICD", "implantable cardioverter defibrillator", "implantable cardioverter-defibrillator", "leadless pacemaker", "Pacemaker", "S-ICD", "subcutaneous implantable cardioverter defibrillator", "subcutaneous implantable cardioverter-defibrillator"]	["aunt", "auntie", "bro", "brother", "brother in law", "brother-in-law", "caregiver", "carer", "child", "cousin", "dad", "daughter", "daughter in law", "daughter-in-law", "father", "father in law", "father-in-law", "fiance", "fiancé", "fiancee", "fiancée", "friend", "girlfriend", "gramps", "gran", "granddaughter", "grandfather", "grandma", "grandmother", "grandpa", "grandson", "husband", "mama", "mom", "mother", "mother in law", "mother-in-law", "mum", "neighbour", "nephew", "niece", "other half", "papa", "partner", "sibling", "sis", "sister", "sister in law", "sister-in-law", "son", "son in law", "son-in-law", "spouse", "step brother", "step dad", "step daughter", "step father", "step mom", "step mother", "step sister", "step son", "stepbrother", "step-brother", "stepdad", "step-dad", "stepdaughter", "step-daughter", "stepfather", "step-father", "stepmom", "step-mom", "stepmother", "step-mother", "stepsister", "step-sister", "stepson", "step-son", "uncle", "wife"]

Table S4.

Full Analysis Structure with Occurrence Frequencies Calculated Based on the Total Number of Participants.

Categories	Subcategories	Codes	*n*	% of *N*
Concerns about the health of patients			120	79.5%
	Medical questions about health conditions and CIEDs		87	57.6%
		Wants further explanation of medical conditions and CIEDs	54	35.8%
		Wants further information about medical risks to the patient	28	18.5%
		Asking about CIED and lead replacement	13	8.6%
		Questions about surgeries and recovery	10	6.6%
		Asking for others to interpret medical information and tests	8	5.3%
		Asking if an ICD device will shock the patient	4	2.6%
		Asking about medical risks of not continuing with a CIED	3	2.0%
		Asking about potential damage to the CIED	2	1.3%
		Questions about CIED faults or recalls	2	1.3%
	Concerns about patients' cardiac symptoms		59	39.1%
		Concerned about changes in heart rate and arrhythmia	20	13.2%
		Concerned that CIED has not improved the patient's cardiac condition	17	11.3%
		Concerned about patient's shortness of breath and breathing	16	10.6%
		Concerned about patient's energy level and weakness	15	9.9%
		Concerned about patient's dizziness or syncope	9	6.0%
		Concerned about patient's fluid retention and swelling	9	6.0%
		Concerned about patient's blood pressure changes	6	4.0%
		Concerned about the patient sweating	3	2.0%
		Concerned about patient having artery problems and strokes	2	1.3%
		Concerned about patient's low blood oxygen	2	1.3%

		Concerned that the patient has had or will have a heart attack	2	1.3%
		Concerned about the patient’s body temperature changes	1	0.7%
		Concerned about the patient’s experiences of chest pain	1	0.7%
		Concerned about patient yawning	1	0.7%
	Concerns about patients’ CIEDs		30	19.9%
		Concerned about CIED battery life	7	4.6%
		Concerned about patient getting ICD shocks	6	4.0%
		Concerned about the patient dependent on the CIED	4	2.6%
		Concerned that the leads or device has been damaged or displaced	4	2.6%
		Concerned about CIED settings and software	3	2.0%
		Concerned about abandoned CIEDs or leads	2	1.3%
		Concerned about CIED-related infections	2	1.3%
		Concerned about the CIED protruding	2	1.3%
		Concerned about the CIED making sounds	2	1.3%
		Concerned about the CIED malfunctioning	2	1.3%
		Concerned the CIED was unnecessary	1	0.7%
	General concerns about patients’ health		29	19.2%
		Concerned about patient’s quality of life	11	7.3%
		Concerned about patient’s experiences of pain	8	5.3%
		Concerned about medications and side effects	5	3.3%
		Concerned about the patient experiencing non-CIED related infections	4	2.6%
		Concerned about changes in the patient’s weight	4	2.6%
		Concerned about the patient going through menopause	1	0.7%
	Concerns about patients’ mental health		17	11.3%
		Concerned about the patient experiencing anxiety or fear	9	6.0%
		Concerned about the patient experiencing PTSD symptoms	4	2.6%
		Concerned patient is losing their cognitive abilities	3	2.0%
		Concerned about the patient being depressed	2	1.3%

		Concerned about the patient being stressed	2	1.3%
Managing and coping with patients' lives and deaths			114	75.5%
	Questions and Advice Sought on Managing Patients' Care and Lives		60	39.7%
		Seeking advice on how to manage or support the patient	32	21.2%
		Asking about what activities patients can do and items they can use	20	13.2%
		Asking about devices to monitor the patient	8	5.3%
		Asking if the patient should go to the emergency room	8	5.3%
		Asking about emergency alert or identification systems	3	2.0%
		Asking how to cope with the negative psychological experiences of the caregiver and patient	3	2.0%
		Asking about being a parent to a CIED patient	2	1.3%
		Asking about CIED remote monitoring	2	1.3%
		Asking about expectations after ICD discharge	1	0.7%
		Asking what to do if patient has a heart attack	1	0.7%
	Difficulties with maintaining patient and caregiver lifestyles		44	29.1%
		Concerned about the patient and electromagnetic interference	9	6.0%
		Concerned about the patient and caregiver traveling	8	5.3%
		Caregiver concerned about the patient living independently	7	4.6%
		Concerned about disturbances in patient's sleep patterns	6	4.0%
		Concerned about patient diet and maintaining a diet	6	4.0%
		Concerned about the life of the patient and caregiver changing	5	3.3%
		Concerned that the CIED limits the patient from doing physical activities	5	3.3%
		Trying to keep the patient's life as normal as possible	5	3.3%
		Concerned about the patient returning to work	2	1.3%
		Concerned about the patient driving	2	1.3%
		Concerned about patient flying with a device	2	1.3%
		Concerned about the patient getting body modifications	1	0.7%

		Concerned about impacts of weather on the patient	1	0.7%
		Concerned about the patient going to high altitude places	1	0.7%
		Concerned about the patient's inactive lifestyle	1	0.7%
		Concerned about the patient as they live rurally	1	0.7%
		Trying to live life to the fullest	1	0.7%
		Trying to maintain a healthy lifestyle	1	0.7%
	Difficulties with patients' surgeries and recovery		28	18.5%
		Concerned about the patient receiving surgeries and potential risks	12	7.9%
		Concerned about the sensitivity and changes in the surgical site of the CIED	8	5.3%
		Concerned about the patient recovering from surgeries	4	2.6%
		Concerned about pain at the surgery site	2	1.3%
		Concerned about possible allergic reaction to the CIED	2	1.3%
		Worried that CIED was not implanted correctly	2	1.3%
		Concerned about patient getting a mechanical heart valve replacement	1	0.7%
		Concerned about patient getting a temporary external pacemaker	1	0.7%
		Concerned that device surgery caused a heart attack	1	0.7%
		Concerned that they started treating the condition too late	1	0.7%
		Previous surgery was horrible for patient and caregiver	1	0.7%
	Questions about death and dying		30	19.9%
		Asking how to prepare for the death of the patient	8	5.3%
		Asking if and when the patient will die	8	5.3%
		Questions about CIED deactivation and removal	7	4.6%
		Wanting an explanation for patient's death	6	4.0%
		Asking subreddit if condition will decrease patient's lifespan	4	2.6%
		Asking if they should travel to see their dying family member	2	1.3%
		Asking about survival time after CIED deactivation	1	0.7%
		Asking if family members should come see the patient	1	0.7%

	Difficulties dealing with the death of patients		26	17.2%
		Worried that the patient will die	16	10.6%
		Concerned about preparing for the death of the patient	4	2.6%
		Wants to provide the patient with a peaceful death	3	2.0%
		Struggling with the fact patients could have died	2	1.3%
		Caregiver is struggling because they are worried about patient death	1	0.7%
		Concerned about the CIED being deactivated prematurely	1	0.7%
		Difficulties as caregiver has prior trauma from a family death	1	0.7%
		Feels like explanation of patient death was oversimplified	1	0.7%
		Patient believes they are dying	1	0.7%
		States that it is hard to deal with death as patient looked fit and healthy	1	0.7%
	The need to monitor patients and prepare for emergencies		16	10.6%
		Concerned about being prepared for medical emergencies	6	4.0%
		Concerned about getting smartwatch or pulse monitor for the patient	6	4.0%
		Concerned about getting medical alert bracelets for patient	2	1.3%
		Concerned about CIED remote monitoring of the patient	2	1.3%
		Concerned about blood pressure monitors	1	0.7%
		Concerned about getting new cell phone	1	0.7%
		Concerned about life alert systems	1	0.7%
		Wants a tool to interrogate patient’s CIED for caregivers	1	0.7%
The difficulties of being a caregiver			94	62.3%
	Using social media for social support		63	41.7%
		Wants to hear about the experiences of other caregivers and patients	38	25.2%
		Seeking reassurance from other users	15	9.9%
		Coming to the platform as they can’t find appropriate information elsewhere	9	6.0%
		Looking for clear and straightforward answers	6	4.0%
		Thankful for stories and advice shared on the platform	6	4.0%

		Sharing information and stories to inform other caregivers and patients	3	2.0%
		Sharing stories to give other caregivers and patients hope	3	2.0%
		Seeking advice on how to cope with mental stress of helping the patient	2	1.3%
		Sharing how they feel about being a caregiver	2	1.3%
		Patient wants advice but caregiver doesn't know what to say	1	0.7%
		Reading the stories of other patients and caregivers gives them hope	1	0.7%
	Negative mental health experiences of caregivers		38	25.2%
		Caregiver is experiencing fear due to the situation	14	9.3%
		Caregiver is emotionally upset due to the situation	7	4.6%
		Caregiver is experiencing anxiety due to the situation	7	4.6%
		Caregiver is experiencing stress due to the situation	6	4.0%
		Caregiver is distressed from seeing the patient being ill	5	3.3%
		Finds it difficult to deal with situation as patient was previously healthy	3	2.0%
		Caregiver feels lost facing situation	2	1.3%
		Caregiver has trauma due to the situation	2	1.3%
		Caregiver is experiencing depression due to the situation	2	1.3%
		Situation was a shock to caregiver	2	1.3%
	Difficulties Arising from Patients' Behaviours		18	11.9%
		Cannot trust the patient to tell the truth or accurately relay symptoms	8	5.3%
		Patient does not want to see healthcare providers or get treatment	7	4.6%
		Patient resists advice from healthcare providers	3	2.0%
		Difficulties as caregiver does not want to upset the patient	2	1.3%
		Caregiver feels bad for pushing patient to get treatment	1	0.7%
		Risk information makes patient nervous	1	0.7%
		Worried about patient learning new things	1	0.7%
	Difficulties with the family		10	6.6%
		Difficulties dealing with other family members or caregivers	5	3.3%
		Worried about patient and caregivers combined future as a family	2	1.3%

		Worried about the future for their child with a device	2	1.3%
		Difficulties from temporary living situation with family	1	0.7%
		Patient condition is making it difficult for the family	1	0.7%
Engagement with healthcare			62	41.1%
	Questions and advice sought about healthcare		35	23.2%
		Questions about the practices and decisions of healthcare providers	22	14.6%
		Asking about second opinions or alternative options for the patients care	6	4.0%
		Directly asking others to explain patient's condition given poor communication from healthcare providers	5	3.3%
		Asking what healthcare providers patients need to see	4	2.6%
		Asking about what medical tests should be conducted	3	2.0%
		Asking if they should advocate for patient	2	1.3%
		Asking about appropriate appointments for the patient	1	0.7%
		Asking if they should get legal advice	1	0.7%
	Negative experiences with healthcare providers		32	21.2%
		Concerned that healthcare providers have not acted in best interest of the patient	16	10.6%
		Concerned that healthcare providers are dismissing concerns	12	7.9%
		Experienced difficulties due to poor communication from healthcare providers	8	5.3%
		Concerned that healthcare providers' actions have hurt the patient	7	4.6%
		Concerned about healthcare providers' eagerness to put a specific device in	2	1.3%
		Struggles to trust healthcare providers due to prior experiences	2	1.3%
		Concerned about conflicting opinions between healthcare providers	1	0.7%
		Concerned that the patient will be dismissed by healthcare providers as they are a person of colour	1	0.7%
	Difficulties with patient healthcare		16	10.6%
		Difficult with access to healthcare	5	3.3%

		Concerned about the accuracy of medical tests	2	1.3%
		Difficult accessing healthcare due to long travel time	2	1.3%
		Wants the best treatment possible for the patient	2	1.3%
		Worried about financial cost and difficulties with insurance	2	1.3%
		Difficult to access doctors as patient lives rurally	1	0.7%
		Wants holistic care for the patient	1	0.7%
		Worried about the safety of MRI scans	1	0.7%
		Worried about wait time to see the doctor	1	0.7%
		Worried patient cannot advocate for themself	1	0.7%
	Positive experiences with healthcare		11	7.3%
		Thankful for and happy with healthcare providers	5	3.3%
		Happy that healthcare and device saved the patient's life	4	2.6%
		Believes the healthcare providers are doing their best	1	0.7%
		Happy to be rid of the life vest	1	0.7%

Note. Occurrence frequencies calculated based on the total number of participants (*N* = 151) with counts and percentages representing the number of participants to which each analytical strata (category, subcategory, or code) applied.

Figure S1.

Qualitative Research Approach Map of the Study.

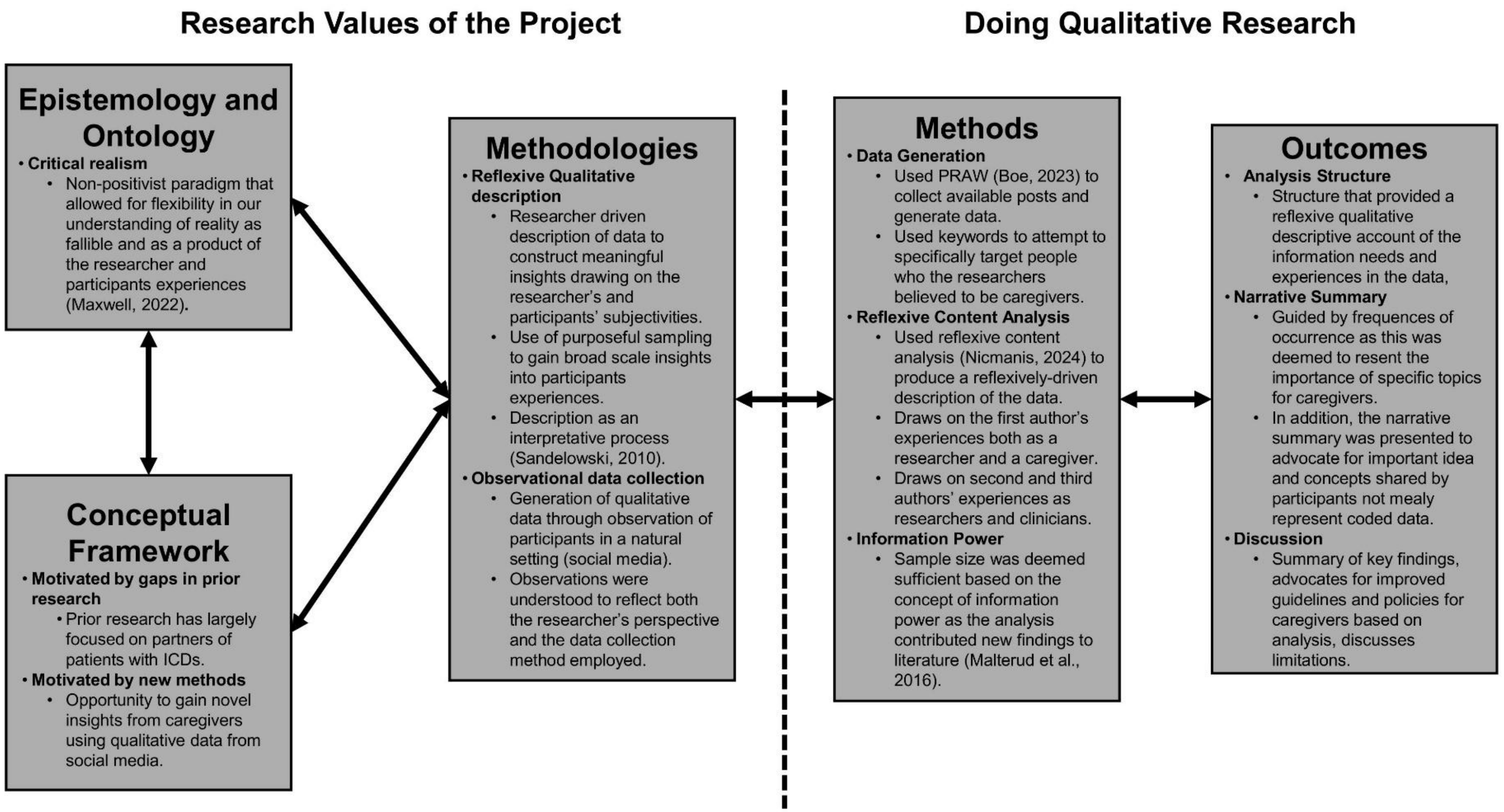

Note: Citations used: Boe, 2023; Malterud et al., 2016; Maxwell, 2022; Nicmanis, 2024; Sandelowski, 2010

References

Boe, B. (2023). *PRAW 7.7.1 Documentation*. https://praw.readthedocs.io/en/stable/#

Malterud, K., Siersma, V. D., & Guassora, A. D. (2016). Sample Size in Qualitative Interview Studies: Guided by Information Power. *Qualitative Health Research*, *26*(13), 1753–1760. https://doi.org/10.1177/1049732315617444

Maxwell, J. A. (2022). Critical realism as a stance for designing qualitative research. In U. Flick (Ed.), *The SAGE handbook of qualitative research design* (Vol. 1, pp. 142–155). SAGE.

Nicmanis, M. (2024). Reflexive content analysis: An approach to qualitative data analysis, reduction, and description. *International Journal of Qualitative Methods*, *23*, Article 16094069241236603. https://doi.org/10.1177/16094069241236603

O'Brien, B. C., Harris, I. B., Beckman, T. J., Reed, D. A., & Cook, D. A. (2014). Standards for Reporting Qualitative Research: A synthesis of recommendations. *Academic Medicine*, *89*(9), 1245–1251. https://doi.org/10.1097/ACM.0000000000000388

Sandelowski, M. (2010). What's in a name? Qualitative description revisited. *Research in Nursing & Health*, *33*(1), 77–84. https://doi.org/10.1002/nur.20362
